# Eukaryotic translation initiation factor 4A1 in the pathogenesis and treatment of cancers

**DOI:** 10.3389/fmolb.2023.1289650

**Published:** 2023-11-09

**Authors:** Jinghong Huang, Lei Zhang, Rui Yang, Lixia Yao, Jinming Gou, Dongdong Cao, Zeming Pan, Dongmei Li, Yuanming Pan, Wei Zhang

**Affiliations:** ^1^ Key Laboratory of Xinjiang Endemic and Ethnic Diseases, School of Medicine, Shihezi University, Shihezi, Xinjiang, China; ^2^ Clinical Laboratory, First Affiliated Hospital of Shihezi University, Shihezi, Xinjiang, China; ^3^ Troops of the People’s Liberation Army, Urumqi, Xinjiang, China; ^4^ Cancer Research Center, Beijing Chest Hospital, Beijing Tuberculosis and Thoracic Tumor Research Institute, Capital Medical University, Beijing, China; ^5^ Shihezi People’s Hospital, Shihezi, Xinjiang, China

**Keywords:** eukaryotic translation initiation factor 4A1, human cancer, clinicopathologic features, biomarkers, inhibitors

## Abstract

Abnormal translate regulation is an important phenomenon in cancer initiation and progression. Eukaryotic translation initiation factor 4A1 (eIF4A1) protein is an ATP-dependent Ribonucleic Acid (RNA) helicase, which is essential for translation and has bidirectional RNA unwinders function. In this review, we discuss the levels of expression, regulatory mechanisms and protein functions of eIF4A1 in different human tumors. eIF4A1 is often involved as a target of microRNAs or long non-coding RNAs during the epithelial-mesenchymal transition, associating with the proliferation and metastasis of tumor cells. eIF4A1 protein exhibits the promising biomarker for rapid diagnosis of pre-cancer lesions, histological phenotypes, clinical staging diagnosis and outcome prediction, which provides a novel strategy for precise medical care and target therapy for patients with tumors at the same time, relevant small molecule inhibitors have also been applied in clinical practice, providing reliable theoretical support and clinical basis for the development of this gene target.

## 1 Introduction

According to the recent statistical analysis of global cancer types and cancer incidence in various countries, cancer is one of the most important diseases affecting human beings, causing serious physical damage and psychological stress to patients (BRAY et al., 2018). Cancer is a disease that occurs in the context of a complex interplay of multiple factors. Cancer occurs under the complex regulation of multiple factors, and the final manifestation *in vivo* is the alteration of the expression of the relevant oncoproteins, which is strictly controlled during the translation of messenger RNA (mRNA).

mRNA translation dysregulation is one of the most important factors in the predisposition of cancer (WALDRON et al., 2019). mRNA translation is a complex process that includes an initiation step, an extension step, and a termination step (DEVER and GREEN, 2012). The rate-limiting step of most protein synthesis occurs at the initiation stage, and its aberrant regulation greatly contributes to translational reprogramming, which characterizes cancer cells, such as abnormal proliferation and chemoresistance (JACKSON et al., 2010). The translation initiation program in cancer cells maintains their malignant capacity in tumor progression and metastasis. Downregulation of translational capacity, particularly through inhibition of translation initiation will result in diminished tumor migration and invasiveness (MANIER et al., 2017).

Eukaryotic Translation Initiation Factor (eIF) have multiple functions in these processes: They can act as RNA chaperones, ATP-dependent RNA helicases, as RNPases by mediating RNA-protein association and dissociation or as co-activators and co-repressors of translation (SCHUTZ et al., 2010). There is substantial evidence that eIF is strongly associated with poor prognosis and resistance to chemotherapy and targeted therapy in many cancers, including leukemia (WOLFE et al., 2014).

Members of the eukaryotic translation initiation factor 4 A family are required for the process of translation initiation, and they are also prototypical of members of the dead-box (DEAD-box) family. The DEAD-box family acts as a helicase that unwinds RNAs, separating base paired RNA strands bound by hydrogen bonds from one another and removing secondary structures in RNA, so that they can be ligated by RNA enzymes, a change that is dependent on the supply of energy from ATP. The eIF4A structural domain was the first DEAD-box protein structure to be identified. Box protein structure with RecA-like folding (binding sites for nucleotides) and interactions between conserved motifs within the structural domains (LINDER, 2006). The eIF4A family includes the following isoforms: eIF4A1, eIF4A2, and eIF4A3 (XUE et al., 2021). Generally, eIF4A1 and eIF4A2 are mainly located in the cytoplasm and are more abundant in eIF4A1 relative to eIF4A2, while eIF4A3 is mainly located in the nucleus (LU et al., 2014). eIF4A1 and eIF4A2 are mainly involved in the initiation of mRNA translation, whereas eIF4A3 proteins mainly play a role in RNA metabolism, including mRNA localization, export, and coupling of mRNA’s splicing to translation (MAZLOOMIAN et al., 2019).

Recent studies have shown that eIF4A1 as the ATP-dependent RNA helicase has low activity. Its RNA unwinding activity is coupled to a conformational cycle in which eIF4A alternates between an open conformation with a wide cleft between its two RecA domains (SUN et al., 2014), and a closed conformation in the presence of ATP and RNA, in which the two RecA domains interact with each other and with bound ATP and RNA. Formation of the closed state is linked to duplex destabilization (ANDREOU and KLOSTERMEIER, 2014). Upon ATP hydrolysis and phosphate release, eIF4A assumes an open conformation and disengages from the RNA. Binding of free eIF4A to ATP or RNA is not ordered but is coupled (PELLETIER and SONENBERG, 2019). The preferred substrate for eIF4A1 is a single-stranded poly-purine RNA. RNA fragments as short as 4 nucleotides can stimulate the ATPase activity of free eIF4A, but fragments of 15–20 nucleotides are optimal (PECK and HERSCHLAG, 1999) eIF4A1 binds to PKP1 in the eIF4F cap-binding complex and is stimulated by PKP1 to promote ATPase activity, thereby increasing the translation rate of mRNAs (WOLF et al., 2010). eIF4A1 can also be deubiquitinated by interacting with ubiquitin-specific peptidase 15 (USP15), which promotes the self-stabilization of eIF4A1, thereby increasing the translation efficiency of mRNAs (ZHAO et al., 2020). However, MA et al. (2019) found that inhibition of PHGDH reduces its interaction with eIF4A1, decreases eIF4A1 activity, and blocks the formation of the translation initiation complex eIF4F, thereby preventing the translation of the entire mRNA process.

The ATP-dependent RNA helicases eIF4A1 plays a crucial role in various cancers in humans (WOLF et al., 2010) The expression level of eIF4A1 varies in different types of malignant tumors (LIN et al., 2018). With the understanding and deepening of the regulatory mechanism of eIF4A1, we found that eIF4A1 can be one of the potential points of action and biomarkers for cancer diagnosis and therapy (HAN et al., 2022). In this paper, we will discuss the regulatory mechanism and biological function of eIF4A1 during mRNA translation. In addition, this review also discusses the role of eIF4A1 in the process of tumor proliferation and metastasis, and existing inhibitors, suggests the possibility of using them as a potential point of action and biomarker for cancer diagnosis, treatment and prognosis.

## 2 Regulatory mechanisms of eIF4A1

eIF4A1 is an important component of eIF4F, and the eIF4F cap-binding complex consists of the eIF4A1 translation initiation factor, eIF4G scaffolding protein, and eIF4E m7 G-binding protein (JACKSON et al., 2010). Many eIFs are assembled with small (40S) ribosomal subunits to form a 43S pre initiation complex, which scans the first AUG codon on mRNA and then binds to the large (60S) subunit to form an active 80S ribosome on this initiation codon (IWASAKI et al., 2019). Through the eIF4F complex, eIF4A1 is involved in two main translation steps: loading mRNA onto the 43S pre-initiation-complex and translocating it along the 5'UTR to the translation initiation point (SCHMIDT et al., 2023). The loading function requires only eIF4A1’s ATPase activity (WANG et al., 2022). eIF4A1 relies on ATP binding to single stranded RNA (AMBARU et al., 2022), unlocking the double stranded region in RNA (WILCZYNSKA et al., 2019). In addition, eIF4A1 itself is a weak helicase, and the dissociation efficiency achieved by eIF4A1 is significantly improved by forming a complex of eIF4A1 and cofactor proteins eIF4G, eIF4B, and eIF4H that synergistically regulate the conformational cycle of eIF4A1 (GARCíA-GARCíA et al., 2015). The translation initiation factors eIF4B and eIF4G jointly stimulate the weak intrinsic RNA-dependent ATPase and ATP-dependent RNA helicase activities of yeast and human eIF4A (OZES et al., 2011) through modulation of the eIF4A conformational cycle. In the presence of eIF4G, eIF4A alternates between a half open conformation, stabilized by binding of eIF4G to both RecA domains of eIF4A, and the closed state (HARMS et al., 2014). eIF4B binds to eIF4A through its 7-repeats domain. Binding of eIF4B to eIF4A further accelerates closing when eIF4G is present, and thus causes an additional shift of the conformational equilibrium of eIF4A toward the closed state (JOYCE et al., 2017). So its unwinds function is largely dependent on the stimulation of its binding partner, whereas the binding of single-stranded RNA can largely stimulate the activity of eIF4A (SHEN and PELLETIER, 2020). eIF4B and eIF4H also stabilize partially unwound substrates and/or prevent mRNA reannealing, activities that further facilitate RNA restructuring during initiation.

As an important translation initiation factor, eIF4A1 loads all mRNAs and binds to ribosomes. However, recent research results indicate that RNA is also involved in influencing the function of eIF4A1. For example, the catalytic activity of the complex eIF4A-eIF4B-eIF4G can be increased by the length of single stranded RNA (ANDREOU et al., 2019), However, the role of RNA in regulating the function of eIF4A1 has not been thoroughly studied.

Previous studies have demonstrated that translational dysregulation is an important step in tumorigenesis and progression that directly controls selective translation and protein synthesis of cancer genes (WALDRON et al., 2019). The eIF4F translation initiation complex, under the regulation of the PI3K/Akt/mTOR signaling pathway, the mitogen-activated protein kinase signaling pathway, and the cysteine-dependent apoptosis pathway, serves as a key node for translation initiation and controls the translation initiation phase of mRNAs of many oncogenes (LIN et al., 2008; BLAGDEN and WILLIS, 2011). As an important component of eIF4F, eIF4A1 plays an important role in the process of gastric carcinogenesis and development and epithelial mesenchymal transition in gastric cancer, and recent studies have shown that the expression of eIF4A1 in cancers such as gastric cancer, colorectal cancer, cervical cancer, breast cancer, and melanoma exhibits abnormalities (LIANG et al., 2014; MODELSKA et al., 2015; JOYCE et al., 2017; GAO et al., 2020; CHEN et al., 2021a; SOYLEMEZ et al., 2021). Mutations in eIF4A1 lead to translational repression (WILCZYNSKA et al., 2019). Free eIF4A1 is regulated by Programmed Cell Death 4 (PDCD4), which binds eIF4A, blocks formation of the closed conformation. And the level of eIF4A1 itself is regulated by mTOR and the carcinogen miR-21 (ASANGANI et al., 2008).

## 3 Expression of eIF4A1 in different tumors

eIF4A1 is dysregulated and aberrantly expressed in many different tumor tissues (LIN et al., 2018), although the exact role of eIF4A1 in tumorigenesis and development is unclear, it may be associated with abnormal RNA unwinds function and lead to aberrant expression of proteins formed by aberrant RNA translation (LOH et al., 2009).

### 3.1 Expression of eIF4A1 in gastric cancer

Gao et al. (2020) used Gene Expression Omnibus (GEO) to detect the mRNA level of EIF4A1 expression in gastric cancer (GC) tissues, and the data showed that eIF4A1 expression was significantly upregulated in gastric cancer compared with that in adjacent normal tissues. Furthermore, IHC results of GC patients showed that eIF4A1 protein levels were generally elevated in GC tissues compared with normal ones (56.5%, 108/191) (GAO et al., 2020). Wei et al. also found that the protein levels of eIF4A1 expression were significantly upregulated in 74 clinical cancer samples and its control samples (58.1%, 58/74) (WEI et al., 2019). Furthermore, overexpression of eIF4A1 was significantly correlated with advanced tumor metastasis, epithelial mesenchymal transition, poor tumor differentiation and poor prognosis in GC patients (GAO et al., 2020).

### 3.2 Expression of eIF4A1 in colorectal cancer

Li et al. found that immunohistochemical staining in colorectal cancer patients showed that eIF4A1 was highly expressed in 86% (44/51) of primary colorectal cancer tissues (LI et al., 2017). However, Zafer et al. found that eIF4A1 was highly expressed in stage II colorectal cancer tissues and lowly expressed in stage I, III, and IV colorectal cancer tissues, and that eIF4A1 was highly expressed in the peripheral blood of patients with stage I, II, and III colorectal cancer, but lowly expressed in patients with stage IV colorectal cancer (SOYLEMEZ et al., 2021). Yang et al. reported that eIF4A1 is recruited by Long noncoding RNA (LncRNA) MAPKAPK5-AS1 to promote translation of MAPK-activated protein kinase 5 in colorectal cancer cells (YANG et al., 2020).

### 3.3 Expression of eIF4A1 in cervical cancer

Liang et al. found that overexpression of eIF4A1 was detected in 83.9% of cervical cancer tissues and that overexpression of eIF4A1 was associated with advanced tumor proliferation, lymph node metastasis, squamous cell in patients with cervical cancer, before and after brachytherapy by using immunohistochemistry in 35 cases of normal cervical tissues, 87 cases of cervical cancer tissues without surgical treatment, and 50 pairs of cervical cancer tissues histology, deep mesenchymal invasion, and poor prognosis were significantly correlated (LIANG et al., 2014). They also found that silencing eIF4A1 can increase the radiosensitivity of cervical cancer, leading to delayed repair of radiation-induced DNA double strand breaks (LIANG et al., 2014)

### 3.4 Expression of eIF4A1 in breast cancer

The study by Modelska et al. found that upregulation of eIF4A1 expression in estrogen receptor-negative breast cancers were associated with higher histological grades by immunohistochemical testing of tissue microarrays from approximately 4,000 patients and by post-statistical analysis of the patients’ cancer grades (89.5%), and that eIF4A1 is involved in dysregulation of the mRNA translation process through pro-carcinogenic signaling, which contributes to breast cancer in the generation of malignant phenotype, suggesting that eIF4A1 can be used as a biomarker to predict the prognosis of breast cancer patients (MODELSKA et al., 2015).

### 3.5 Expression of eIF4A1 in melanoma

Eberle et al. found that eIF4A1 was consistently overexpressed in melanoma cells and discovered that eIF4A1 aided melanocytoma proliferation, whereas inhibition of endogenous eIF4A1 expression suppressed the value-added migratory and invasive abilities of melanocytomas (64.2%) (EBERLE et al., 2002).

### 3.6 Expression of eIF4A1 in other tumors

Zhao et al. reported that low levels of PDED4 and high levels of eIF4A1 predicted poorer differentiation and higher recurrence rates after surgery for oral squamous carcinomas, suggesting that these proteins are significant independent risk factors for such cancers(47.8%, 33/69) (ZHAO et al., 2019). Wang et al. found that for prostate cancer cells, elevated mRNA levels of eIF4A1 correlated with DNA hypomethylation levels on CpG-rich eIF4A1 islands, eIF4A1 translation products were epigenetically regulated through DNA methylation, and eIF4A1 exerted its oncogenic effects through BRD2 signaling (WANG et al., 2022). Similarly, Zhao et al. found that in Myc-amplified G3-type medulloblastoma (G3-MB), eIF4A1 was highly expressed and positively correlated with Myc expression, and that inhibiting eIF4A1 expression could effectively inhibit Myc expression at the translational level, and through this process, promote apoptosis of G3-MB cells and inhibit G3-MG cell proliferation to block the growth of tumor cells (ZHAO et al., 2020). Recently, in a study on brain gliomas (KRASSNIG et al., 2021) and endometrial cancer (LOMNYTSKA et al., 2012) and human cytomegalovirus (QI et al., 2013), there were also showing that eIF4A1 has significantly higher expression levels in different tumors and functions as a tumor promoter.

## 4 Biological functions of eIF4A1 protein in different tumors

About the biological functions of eIF4A1 protein, Wolfe et al. reported that an eIF4A-dependent mechanism of translational control that is encoded in the 5′-UTR of susceptible transcripts, including many oncogenes and transcriptional regulators (for example, Myc, Myb, Notch, Cdk6, Bcl2, and others). Thereby accelerating the progression of Notch-driven T-cell acute lymphoblastic leukemia (WOLFE et al., 2014). Similarly, Cailin et al. found that eIF4A1 is generated by acting on the coding region and 3′-UTR of mRNA to produce an effect on the translational phase of melanoma cells (JOYCE et al., 2017). Li et al. reported that eIF4A1 is a direct target of miR-133a, and miR-133a inhibit colon cancer cells by inhibiting eIF4A1 expression (LI et al., 2017). Similar to the role of miR-133a, miR-1284 can directly inhibit the expression of EMT related genes c-Myc and MMP12 by inhibiting eIF4A1 in gastric cancer (WEI et al., 2019).

Ritesh et al. found that eIF4A1 stimulation by Raf/MAPK/extracellular signaling pathway-regulated kinase signaling significantly promoted the expression of genes associated with the cell cycle and accelerated tumor size in cutaneous squamous cell carcinoma. Combined inhibition of the Raf/MAPK/extracellular signaling-regulated kinase axis and eIF4A1 decreased the 5′-capsule-dependent translational process and attenuated the growth, metastasis, and invasiveness of cutaneous squamous cell carcinoma cells (ZHAO et al., 2019; SRIVASTAVA et al., 2021). Nishida et al. found that eIF4A1 promoted the proliferation of Heat Shock Factor 1 (HSF1) in cells, thereby promoting the proliferation of progenitor cells and leukemia-initiating cells and accelerating leukemogenesis (NISHIDA et al., 2021).

Xu et al. found that eIF4A1 was also involved in the critical steps of platelet healing. SiRNA-USP15 was shown to be involved in platelet healing through promoting eIF4A1 de-ubiquitination enhanced the functional properties of platelet cells to promote wound healing (XU et al., 2021). Zhao et al. reported that in pancreatic cancer cells, eIF4A1 elevated the expression of E-cadherin and N-cadherin through the c-myc/miR-9 axis. eIF4A1 and c-myc promoted epithelial mesenchymal transformation and metastatic ability of pancreatic cancer cells, while eIF4A1 alone upregulation reduced the inhibitory effect of c-myc downregulation on epithelial mesenchymal transformation and metastasis. The eIF4A1 inhibitor Rocaglamide (RocA) and the c-Myc inhibitor Mycro3, alone or in combination, significantly reduced the expression levels of markers of epithelial mesenchymal transition in pancreatic cancer cells (ZHAO et al., 2021).

**FIGURE 1:**
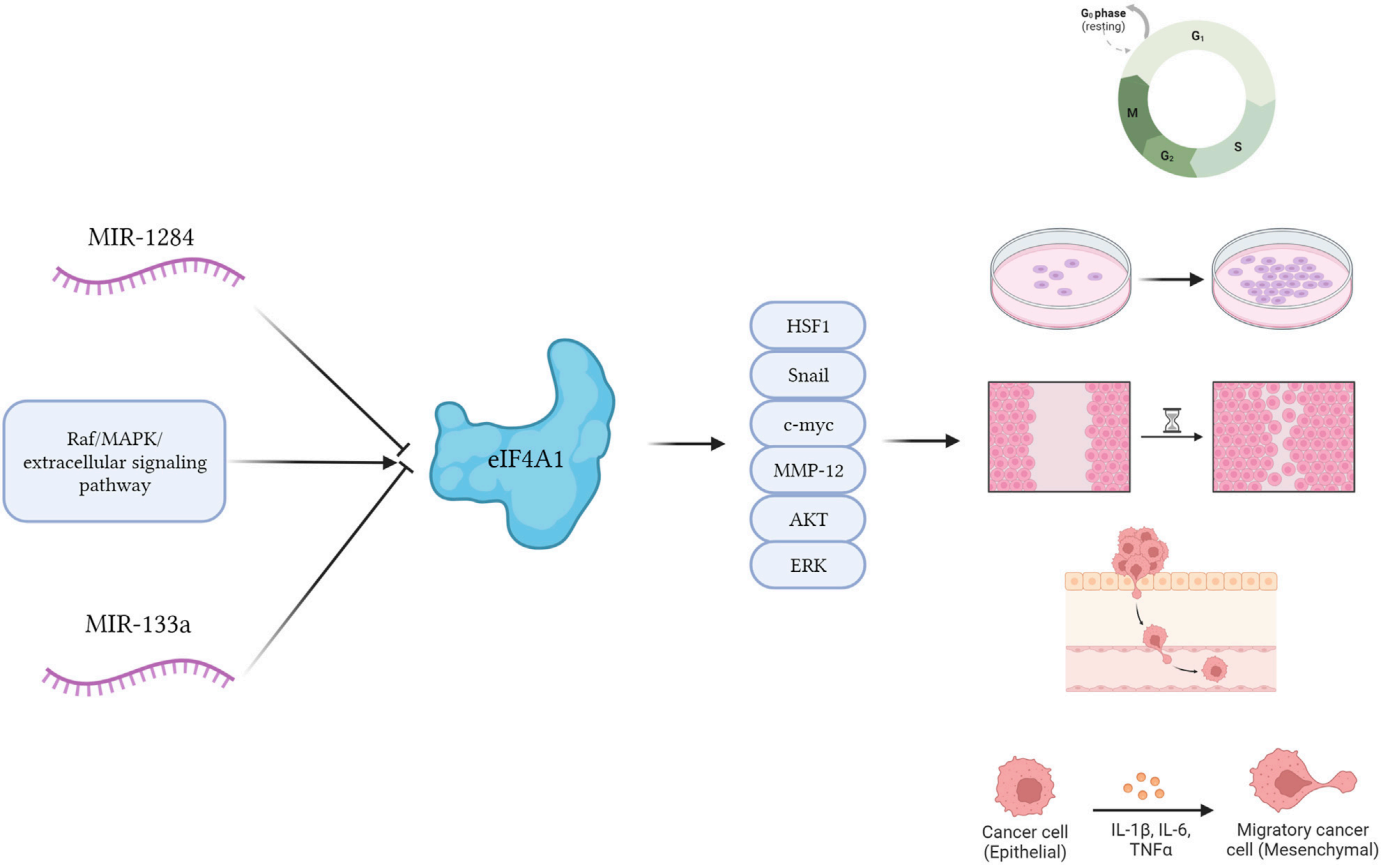
The potential mechanism diagram of eIF4A1. eIF4A1 stimulation by Raf/MAPK/extracellular signaling pathway-regulated kinase signaling significantly promoted cellular behavior such as proliferation, migration, invasion, and EMT of cancer cells through Snail, c-myc, MMPs, AKT and ERK proteins. MiRNAs such as miR-1284 and miR-133a can inhibit the expression of eIF4A1 protein, and these behaviors occur.

Oblinger et al. reported that inhibition of eIF4A1 with the eIF4A1 inhibitor, Cevistro, consistently reduced the expression levels of several cell cycle proteins, Aurora A kinase, and the mitogen-activating enzymes, AKT and ERKs in nerve sheath tumors (OBLINGER et al., 2016). Cevastro treatment significantly inhibited tumor proliferation in nerve sheath tumors. Joyce et al. found that silencing of eIF4A1 in WM858 cells significantly reduced melanoma proliferation and invasion (JOYCE et al., 2017). (Figure 1)

## 5 eIF4A1 inhibitors as the promising drugs in cancer treatment

Based on the above results, it can be concluded that the high expression level of eIF4A1 significantly stimulates the malignant phenotype (proliferation, invasion, migration, and epithelial mesenchymal transition) of cancer cells. Therefore, the upregulation of eIF4A1 seems to have an impact on transformed cells through specific information, making eIF4A1 an attractive target for therapeutic interventions. Several natural compounds have been described as inhibiting cap-dependent translation by specifically inhibiting eIF4A1 activity, including Hippuristanol (CHIO et al., 2016), Pateamine A (NAINENI et al., 2021) and Rocaglates (CHEN et al., 2021b). (Table 1)

**TABLE 1:** Structure and action of different eIF4A1 inhibitors.

Name	Structure	Combination method and action	Ref.
Hippuristanol	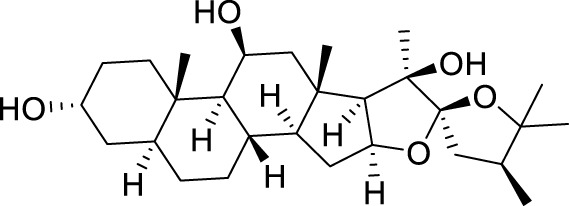	Interact with amino acids present within or adjacent to the motif in the CTD of eIF4A1 and cancels the RNA binding activity of eIF4A1 by locking the helicase in a closed conformation.	(SUN et al., 2014; HOWARD et al., 2020)
Rocaglamide	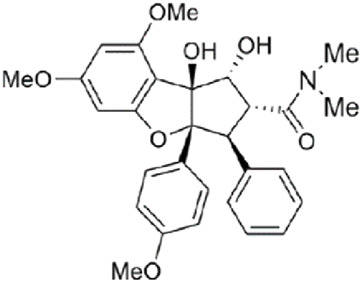	Specifically bind in a cavity formed between human eIF4A1 (at Phe163, Gln195, Asp198, and Ile199) and adjacent purines (A and G). Whenever ATP-bound eIF4A binds to RNA and kinks it to induce unwinding, a bimolecular cavity is formed between the eIF4A NTD and the bent single-stranded RNA. When human eIF4A1 binds to consecutive purine residues, the resultant bimolecular cavity can accommodate Rocaglate or Pateamine A. The complex leading to translation repression.	INGOLIA (2021)
Pateamine A	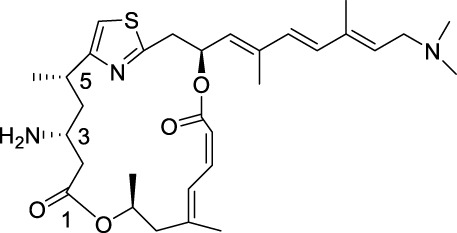		
Elatol	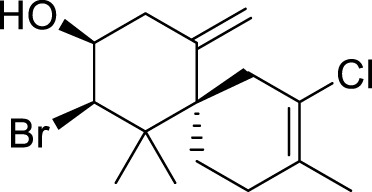	N/A	PETERS et al. (2018)
eFT226	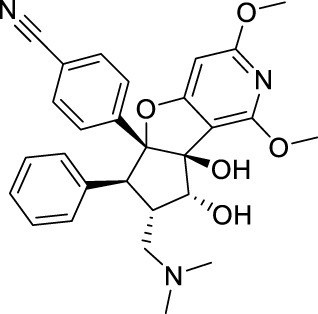	It forming a ternary complex with eIF4A and AGAGAG purine RNA oligonucleotides, preventing eIF4A1 from releasing from the purine RNA motif.	ERNST et al. (2020)

Hippuristanol is a natural product originally isolated from the Isis hippopotamus (CHAO et al., 2005), A type of bamboo coral that cancels the RNA binding activity of eIF4A1 by locking the helicase in a closed conformation (SUN et al., 2014). Hippuristanol has been shown to interact with amino acids present within or adjacent to the motif in the CTD of eIF4A1, This binding site is not conserved in other DEAD box RNA helicases (HOWARD et al., 2020).

Recently, structural analysis of the Rocaglates: eIF4A1: Polypurine RNA complex has shown that Rocaglates, as an eIF4A1 inhibitor, it interacts indispensable with eIF4A1 and two adjacent RNA purine bases (CHU et al., 2019). Rocaglates are a class of artemisinins extracted from plants containing cyclic penta [*b*] benzofuran structures, and are one of the most effective and specific eIF4A inhibitors known (NAINENI et al., 2020). Over 200 natural and synthetic Rocaglates have been described since Rocaglamide A was first isolated from Asian mahogany, the only genus known to produce Rocaglates (GREGER, 2022). Structural analysis of the eIF4A: Rocaglates: Polypurine RNA complex has revealed that Rocaglates specifically bind in a cavity formed between human eIF4A1 (at Phe163, Gln195, Asp198, and Ile199) and adjacent purines (A and G) (IWASAKI et al., 2019).

Similar in function to the complex formed by Rocaglate analogues, the complex formed by Patemine A with eIF4A1, mRNA, and AMPPNP also has the function of inhibiting eIF4A1, although the structures of these two complexes are very different (NAINENI et al., 2021). In addition to overlapping their binding positions on the eIF4A1 protein, Rocaglates and Patemine A also interact directly with RNA. RNA bases are aromatic rings with flat hydrophobic surfaces. Adjacent bases stack with each other in the double helix of RNA (and DNA), burying hydrophobic bases and providing favorable interactions between π-electrons in their aromatic systems. Stacking also contributes greatly to the conformation of single stranded nucleic acids, and RNA binding proteins can interact with RNA in a similar manner through the π-π stacking of amino acid side chains and RNA bases. However, at the curvature of the spine where the dead box protein binds, the base accumulation is disrupted. These proteins completely rely on contact with the sugar phosphate backbone to bind RNA, without interacting with bases to compensate for lost stacking interactions. Bending the bases on both sides forms a hydrophobic pocket that can be occupied by PatemineA or Rocaglates, where the conjugated π-system in these drugs interacts with RNA through stacking (INGOLIA, 2021).

Although the interactions between Patemine A and Rocaglates with RNA are similar, their internal structures are not entirely the same. PatemineA has an extended linear π-conjugated system that can interact well with all four bases. In contrast, the aromatic ring in Rocaglate can only interact well with purines. These molecular differences translate into differences in sequence selectivity. Patemine A stabilizes protein drug RNA complexes that include both pyrimidine and mixed purine pyrimidine RNA sequences, although weaker than purine RNA, while Rocaglates only forms such complexes with purine RNA (IWASAKI et al., 2016). This selectivity extends to the cellular effects of these drugs. PatemineA inhibits the translation of reporters resistant to Rocaglates, which lack a purine sequence in their 50 leading sequences. The difference between PatemineA derivatives and Rocaglates highlights the potential of stacking interactions to provide sequence specificity in nucleic acid binding; This specificity is more related to alkali specific hydrogen bonding (NAINENI et al., 2021). The natural compound Elatol from the ocean also has similar effects to the above two compounds (PETERS et al., 2018).

In addition to natural compounds, some small molecule eIF4A1 inhibitors have also entered our field of vision. EFT226 (Zotatifin) is the first eIF4A inhibitor to enter human clinical trials. This drug was first used in clinical trials of patients with ER^+^ breast cancer, with an expansion cohort for patients with Cyclin D1 alterations. (ERNST et al., 2020). It promotes the binding of specific mRNA sequences with recognition motifs in eIF4A and 5′-UTRs, and interferes with the assembly of eIF4F complexes downstream of mTOR. Its sensitivity is related to mTOR mediated eIF4A activation (THOMPSON et al., 2021). EFT226 inhibits translation initiation by forming a ternary complex with eIF4A and AGAGAG purine RNA oligonucleotides, preventing eIF4A1 from releasing from the purine RNA motif (ERNST et al., 2020). EFT226 treatment downregulates the protein expression of key translation factors Myc and Bcl6, leading to selective gene expression reprogramming, inhibiting cell proliferation, inducing cell death, and thus producing therapeutic effects on various cancer models (THOMPSON et al., 2021). Also, eIF4A inhibitors repress the protein expression of Cyclin proteins including Cyclin D1 and its binding partners CDK4/6. Similarly, Kong et al. showed that Rocaglates can suppress Cyclin-induced feedback to CDK4/6 inhibitors used in lung cancer (KONG et al., 2019).

Furthermore, some effective ingredients in traditional Chinese medicine have also been found to have inhibitory effects on eIF4A1. Berberine is a cyclopentane [b] benzofuran compound found in cactus plants, several of which are used in traditional Chinese medicine. These traditional Chinese medicines are used to treat contusions, coughs, diarrhea, fever, and inflammation (NEBIGIL et al., 2020).

## 6 Conclusion

eIF4A1 is frequently a target of various microRNAs (miRNAs) or LncRNAs and plays a key role in tumor cell proliferation, invasion and metastasis (GINGOLD et al., 2014). Given the importance of the translation process of mRNAs in cancer development, several small molecules have been shown to have antitumor activity by acting on or inhibiting eIF4A1 (STONELEY and WILLIS, 2015). Recent studies have shown that the natural marine products cycloartenol and cycloterpenol can inhibit eIF4A1 and offer promising prospects for cancer therapy (PETERS et al., 2018). In addition, Equine uranol, Cevistrol and Patamine A are all good inhibitors against eIF4A1 (OBLINGER et al., 2016). Rocagrelor has been shown to have potent antitumor activity *in vivo* and *in vitro* by decreasing the cellular translation rate through enhancing the mRNA binding capacity of eIF4A1 and eIF4A2 (CHU et al., 2019). However, inhibitors of eIF4A1 are still in the preclinical research stage, lacking appropriate clinical trial data and clinical evaluation, and their ability to act as antitumor agents still needs to be further explored. In this paper, we review the differential expression and protein functional role of the eIF4A1 in specific tumor types and the regulatory mechanisms, and discuss the relationship between the eIF4A1 and the large number of immune cell infiltration and tumor malignancy, which will provide clues for the next step of research. Our findings confirm the protein functional role and regulatory mechanism of eukaryotic translation initiation factor 4A1 protein in human cancer. And we propose eukaryotic translation initiation factor 4A1 as a target and biomarker for cancer prognosis, diagnosis and treatment.

Currently, the eIF4A1 inhibitor space is still stagnant in the preclinical stage, clearly defined compounds have complex mechanisms and chemical structures. With the progress of current experiments and the extensive application of artificial intelligence in predicting protein spatial configurations and related fields, it is believed that in the near future, more eIF4A1 related inhibitors will enter clinical trials and applications.

## Abbreviations

EIF4A1, Eukaryotic Translation Initiation Factor 4A1; GEO, Gene Expression Omnibus; GC, Gastric Cancer; G3-MB, G3-type medulloblastoma; HSF1, Heat Shock Factor 1; LncRNA, Long noncoding RNA; mRNA, Messenger RNA; MiRNAs, MicroRNAs; PDCD4, Programmed Cell Death 4; RNA, Ribonucleic Acid; USP15, Ubiquitin-Specific Peptidase 15.
