# Supplementary material for: Eukaryotic translation initiation factor 4A1 in the pathogenesis and treatment of cancers
Source: Front Mol Biosci. 2023 Nov 9;10:1289650. doi: 10.3389/fmolb.2023.1289650 (PMC10666758; doi:10.3389/fmolb.2023.1289650)
Supplement: Supplementary file 1 [file Table1.DOCX]

Supplement Table 1. eIF4A1 application in tumors

| **Cancer type** | **Express in tumor** | **Mechanism** | **Prognosis** | **Reference** |
| --- | --- | --- | --- | --- |
| Gastric cancer | High | eIF4A1 can increase the expression of Snail protein, and thus drive the EMT process of gastric cancer cells. MiR-1284 can directly inhibit the expression of EMT related genes c-Myc, MMP12, and Jun by inhibiting eIF4A1 | Poor | ^[27, 35]^ |
| Colorectal cancer | High | MiR-133a can directly inhibit the proliferation of colorectal cancer cells by inhibiting eIF4A1. MK5-AS1 can recruit RBM4 and eIF4A1 to promote the translation of MK5, thereby promoting the proliferation, migration, and invasion of colorectal cancer | Poor | ^[36, 37]^ |
| Cervical cancer | High | Knocking out *EIF4A1* leads to delayed repair of radiation-induced DNA double strand breaks | Poor | ^[29]^ |
| Breast cancer | High | eIF4A1 may participate in the malignant phenotype of breast cancer by unlocking the G-quadruplex structure | Poor | ^[31]^ |
| Melanoma | High | The joint influence of the translation of oncogenes in eIF4A1 melanoma cells is mediated by the coding region and 3 'UTR | Poor | ^[38]^ |
