# Supplementary figures and images for: Eukaryotic translation initiation factor 4A1 in the pathogenesis and treatment of cancers
Source: Front Mol Biosci. 2023 Nov 9;10:1289650. doi: 10.3389/fmolb.2023.1289650 (PMC10666758; doi:10.3389/fmolb.2023.1289650)

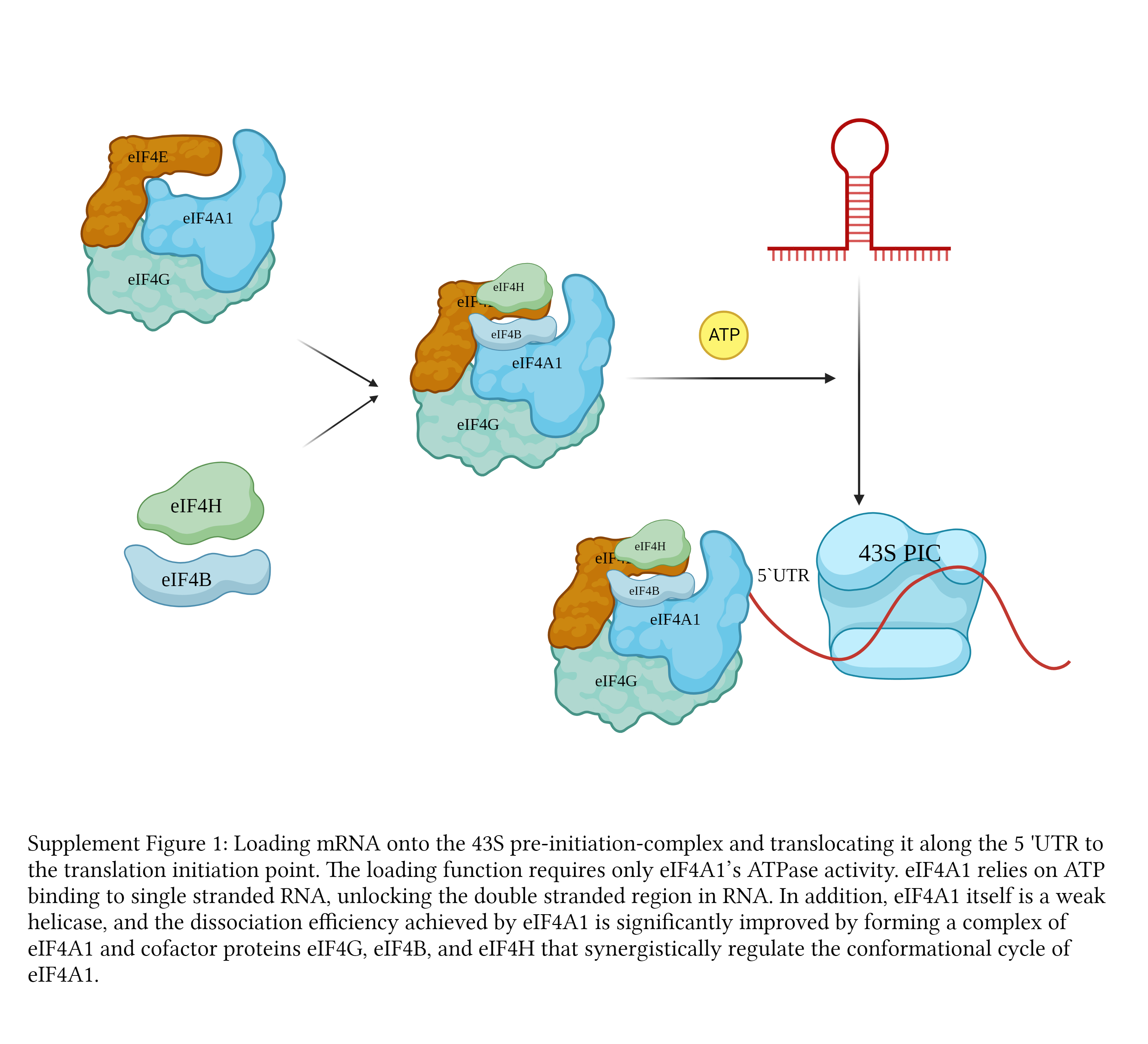

Supplement: Supplementary file 2 [file Image1.JPEG]
